# Estimating uncertainty of alcohol-attributable fractions for infectious and chronic diseases

**DOI:** 10.1186/1471-2288-11-48

**Published:** 2011-04-17

**Authors:** Gerrit Gmel, Kevin D Shield, Hannah Frick, Tara Kehoe, Gerhard Gmel, Jürgen Rehm

**Affiliations:** 1 Centre for Addiction and Mental Health (CAMH) 33 Russell Street, Toronto, Ontario, M5S 2S1, Canada; 2 Ecole Polytechnique Fédérale de Lausanne, Route Cantonale, 1015 Lausanne, Switzerland; 3 Dalla Lana School of Public Health (DLSPH) University of Toronto, 6thFloor, Health Sciences Building 155 College Street, Toronto, Ontario, M5T 3M7, Canada; 4 Ludwig-Maximilians Universität, Geschwister-Scholl-Platz 1, 80539 München, Germany; 5 Department of Statistics, University of Toronto, 100 St. George St., Toronto, Ontario, M5S 3G3, Canada; 6 Addiction Info Suisse, Lausanne, Switzerland; 7 Alcohol Treatment Centre, Lausanne University Hospital CHUV, Mont-Paisible 16, 1011 Lausanne, Switzerland; 8 University of the West of England, Frenchay Campus Coldharbour Lane, Bristol BS16 1QY, UK; 9 Institute for Clinical Psychology and Psychotherapy Technische Universität Dresden, Chemnitzer Str. 46, D-01187 Dresden, Germany

## Abstract

**Background:**

Alcohol is a major risk factor for burden of disease and injuries globally. This paper presents a systematic method to compute the 95% confidence intervals of alcohol-attributable fractions (AAFs) with exposure and risk relations stemming from different sources.

**Methods:**

The computation was based on previous work done on modelling drinking prevalence using the gamma distribution and the inherent properties of this distribution. The Monte Carlo approach was applied to derive the variance for each AAF by generating random sets of all the parameters. A large number of random samples were thus created for each AAF to estimate variances. The derivation of the distributions of the different parameters is presented as well as sensitivity analyses which give an estimation of the number of samples required to determine the variance with predetermined precision, and to determine which parameter had the most impact on the variance of the AAFs.

**Results:**

The analysis of the five Asian regions showed that 150 000 samples gave a sufficiently accurate estimation of the 95% confidence intervals for each disease. The relative risk functions accounted for most of the variance in the majority of cases.

**Conclusions:**

Within reasonable computation time, the method yielded very accurate values for variances of AAFs.

## Background

Alcohol consumption is a major risk factor for burden of disease and injuries globally [1,2] as demonstrated by the Comparative Risk Analyses (CRA) within the Global Burden of Disease and Injury (GBD) Studies [2,3]. To estimate the impact of alcohol consumption on infectious and chronic diseases, alcohol attributable fractions (AAFs) are calculated [4] and applied to the number of deaths or number of incident cases [5].

Up until now, confidence intervals (CIs) have not been presented in the CRA for the estimates of alcohol-attributable health harms. While there are methods to calculate uncertainty around AAFs when both exposure and risk relations are derived from the same cohort [6,7], no such methods exist for the case where both exposure and risk relations stem from two different meta-analyses (for general concerns and considerations see [5,8,9] and the Discussion section below). This article aims to fill this gap, and for the first time will present a method to calculate CIs for the new AAFs modelling methodology used in the 2005 CRA study for chronic diseases by region, sex and age (see [4] for a description of the AAF modelling methodology and [10] for a comparison of the new AAF modelling methodology with previous methods.)

Alcohol is related to many disease categories [5]. Since globally morbidity and mortality can only be reliably estimated for broad disease or injury categories, the GBD is restricted to 126 distinct broad disease or injury categories http://www.globalburden.org/GBD_Study_Operations_Manual_Jan_20_2009.pdf, of which 31 are causally related to alcohol [5]. We will first be using exposure measures and relative risks for disease categories from the 2005 CRA study for which a meta-analysis providing a continuous relative risk function exists to estimate AAFs [5], and then will explain the methodology to construct CIs for these AAFs. This paper will focus on the Asian regions as an illustration of our results. Asia presents an interesting mix of low income and high income regions and allows us to illustrate succinctly our methodology.

## Methods

This method has two main steps: (1) calculation of the AAFs, and (2) calculation of the variance for the AAFs. Information from multiple sources, all of which carries a certain degree of uncertainty, is required in order to calculate the AAFs. This information is outlined below.

### Definition of regions

Regions were defined in accordance with the 2005 GBD study [11]. Countries were grouped into regions which were defined by their geographical location and epidemiological profile which includes child and adult mortality levels and major causes of death. Neither income nor population of the countries in a region had an impact on the grouping. For the purpose of illustrating the method, we restricted the analysis to the five Asian regions containing the countries listed below:

• Asia Pacific, High Income: Brunei Darussalam, Japan, Republic of Korea, Singapore

• Asia Central: Armenia, Azerbaijan, Georgia, Kazakhstan, Kyrgyzstan, Mongolia, Tajikistan, Turkmenistan, Uzbekistan

• Asia East: China, Democratic People's Republic of Korea, Hong Kong, Macao, Taiwan

• Asia South: Afghanistan, Bangladesh, Bhutan, India, Nepal, Pakistan

• Asia Southeast: Cambodia, Christmas Island, Cocos Island, Indonesia, Lao People's Democratic Republic, Malaysia, Maldives, Mauritius, Myanmar, Philippines, Reunion, Seychelles, Sri Lanka, Thailand, Timor Leste, Vietnam

Population estimates for each region by country for 2005 were based on estimates obtained from the 2008 revisions of the United Nations Population Division [12].

### Definition of age categories

Three age categories were used in the CRA study: 15 - 34, 35 - 64 and 65 or greater; we limited our study to the age category of 15 to 34 years. Ages were clustered so that results would be comparable with the 2005 GBD study.

### Measures of alcohol consumption

Adult per capita consumption is calculated by adding together the estimated recorded and unrecorded alcohol consumption [13,14]. The variance of an estimate of recorded consumption was based on estimates from different sources (for example, government data, industry data, Food and Agriculture Organization), which are usually quite similar. The main sources for determining unrecorded consumption are home production, alcohol intended for industrial, technical, and medical uses, and illegal production or importation of alcohol. The variance of an estimate of unrecorded consumption is larger in comparison to that of recorded consumption and there are usually only sparse sources for information on unrecorded consumption which is often based on limited empirical evidence [14,15]. Since uncertainty of unrecorded adult per capita consumption is not provided in the 2005 CRA study, we assumed the standard deviation of unrecorded adult per capita consumption was proportionally five times larger than the standard deviation for recorded adult per capita consumption. The prevalence of lifetime abstainers and former drinkers was estimated from a population-weighted average of surveys in the respective regions by sex and age. Using the proportion of current drinkers we calculated the per capita consumption of alcohol per current drinker, which was used in modelling alcohol consumption. The variance of prevalence can be estimated using a binomial distribution, as illustrated below in the Statistical procedures section.

### Modelling alcohol consumption

Using comparable studies, involving 1001 distributions from 66 countries by sex and age, it can be shown that the distribution of alcohol consumption for the drinking population is modelled best using the gamma distribution [4]. It is well known that population surveys underestimate true consumption, and thus data from surveys have to be triangulated with estimates of adult per capita consumption, which are often based on sales data [4,13]. To be conservative, we assumed that 80% of this registry-based estimate reflected the true adult per capita consumption; this level was chosen to account for the alcohol wasted and not consumed (for example, broken bottles and quantities left over in glasses) and for the underestimation of true consumption in medical epidemiological studies, which were used in the meta-analyses that estimated the relative risk functions. A regression of the above-mentioned studies showed a strong relationship between mean and standard deviation (for men and women, the explained variance of standard deviation was greater than 90%). This relationship allows us to compute the standard deviation of an upshifted distribution very easily. Finally, this method relies on the assumption that the proportion of alcohol consumed by the various sex and age groups derived from surveys is accurate [4].

### Measures of relative risk

**Figure 1:**
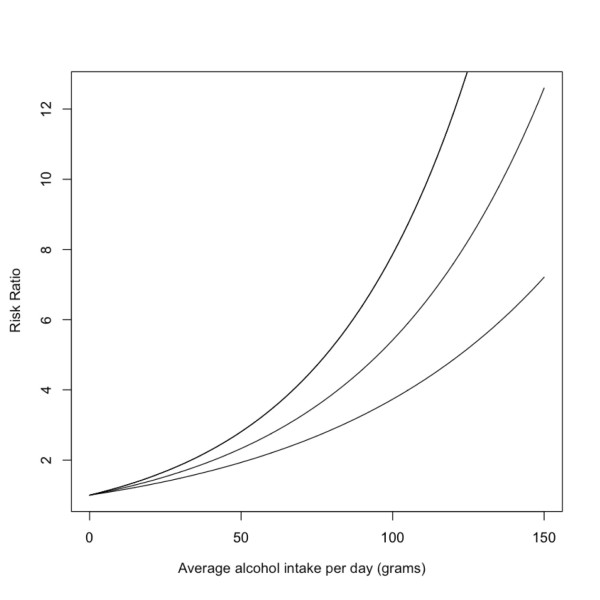
**Risk relationship between average daily intake of alcohol and liver cirrhosis for men**.

**Figure 2:**
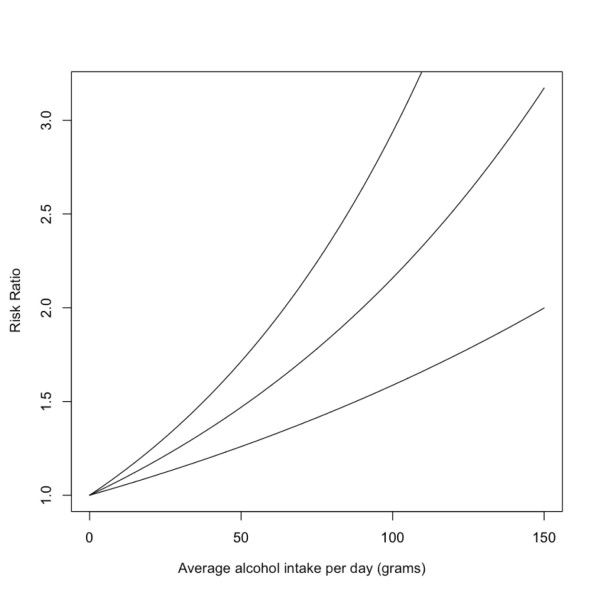
**Risk relationship between average daily intake of alcohol and hemorrhagic stroke for men**.

**Figure 3:**
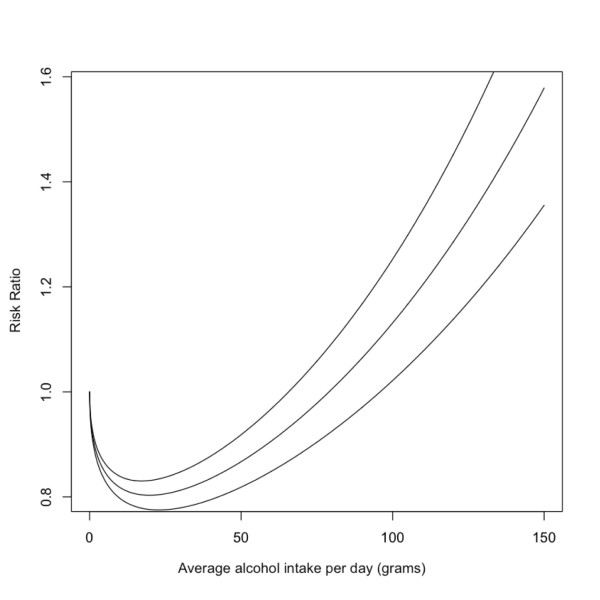
**Risk relationship between average daily intake of alcohol and coronary heart disease for men**.

The relative risk functions for each chronic disease were derived using a series of meta-analyses which used fractional polynomial regression [5] separated by sex and, where possible, by morbidity and mortality (e.g. liver cirrhosis [16] or stroke [17]). The coefficients of each polynomial representing the relative risk function are called beta-coefficients. The uncertainty of the relative risk beta-coefficients is expressed by a covariance matrix (obtained from the meta-analyses). Of the diseases with which alcohol is associated, these associations take one of three forms: 1) exponential, 2) linear, or 3) J-shaped. Figure 1 provides a plot of the relative risk for liver cirrhosis for men as an example of a disease having an exponential relationship with alcohol consumption, figure 2 is a plot of the relative risk for hemorrhagic stroke (mortality) for men which has a linear association with alcohol consumption, and figure 3 outlines the relative risk for coronary heart disease for men as an example of a disease having a j-shaped relationship with alcohol consumption.

#### Step 1: Calculation of the AAF

This step requires calculating the daily consumption AAF estimates, and will be outlined below.

### Alcohol-attributable fractions (AAFs)

The AAF for a given infectious or chronic disease can be expressed as follows [4]:

where P_abs _is the proportion of lifetime abstainers, P_form _is the proportion of former drinkers among the population, and RR_form _is the relative risk of the latter proportion. P(x) represents the prevalence of drinking at level × (in grams per day, modelled by a gamma function), and RR(x) is the relative risk at this level compared to lifetime abstainers. In the CRAs, AAFs are usually calculated separately by sex, age, and sometimes by ethnic groups. In our study of Asian regions, AAFs were computed by region (see below), sex and age.

We did not use this mathematical expression in its original form when estimating the AAFs for several reasons. Firstly, a person whose daily consumption exceeds 150 grams per day is highly unlikely to consume this amount over a long period of time. Therefore, to be conservative, the average daily consumption was truncated at 150 grams per day. Secondly, when there is truncation at 150 grams per day, the gamma distribution needs to be normalized by adding a coefficient in front of the probability density function to ensure that the area under this function will integrate to 1 between 0 and 150 grams of alcohol per day.

#### Step 2: Calculation of the variance of the AAF

This step requires calculating the variance of the AAF estimates with risk data, and will be outlined below.

In order to derive 95% CIs for AAFs, two paths can be taken. The first one consists of deriving the expression for the variance of the AAF by taking into account all the errors of the parameters on which it depends, and subsequently computing the CI for the AAF. This approach, although mathematically accurate, is too complex in our case. Indeed, the AAF depends on the relative risk function, the prevalence of former drinkers and abstainers, and the distribution of consumption among drinkers. Since errors in these values and functions are non-trivial, it is virtually impossible to compute the variance of AAFs algebraically.

The second approach is simpler, but less accurate, and requires more computation. A number (we will call it N for simplicity) of random sets of the lowest level parameters (the parameters from which all other values are derived) are generated, namely the coefficients of the relative risk functions, the adult per capita consumption and the prevalence of former drinkers and lifetime abstainers. Each random set of lowest level parameters will then yield an AAF value for a total of N AAFs for each region, sex, age group and disease. The variance of the N AAFs will approach the true variance as N increases. This corresponds to calculating the variance of an AAF using a Monte Carlo-type method [18].

In order to generate these random samples for each lower level parameter, the distribution, mean and variance of each parameter must be known. The following paragraphs elucidate the methods used to determine the properties of each parameter.

### Statistical procedures

The simulations were implemented in R (version: 2.10.1, refer to "Additional file 1: Example of R - code for simulations" for an example of the code) and the numerical errors inherent in any computational program were neglected (for example, the error (uncertainty) which is added by using numerical integration in calculating the AAFs was not taken into consideration for our variance calculations). The random normal generation of adult per capita consumption for the drinking population sometimes yields values that are negative or zero, which are factually impossible. In these instances, the value was set to 0.001 to symbolize very low consumption. Mathematically, a zero mean consumption would transform the gamma distribution into a Dirac distribution located at 0. In addition, drinkers would have a consumption of 0 grams per day which is not compatible with the definition of current drinkers.

The generation of adult per capita consumption assumes a random normal distribution as we have no information about an alternative distribution. Very low per capita consumption occasionally obtained by the random normal generation caused some additional trouble during the computation. The method used in R to numerically integrate a function results in errors and incorrect results if the corresponding function is either constant or approximately constant. When a gamma distribution has mean values that approach 0, it is spread very little (according to the linear relation between mean and standard deviation). This makes the distribution approximately constant after the initial spike close to the origin. These functions cannot be integrated and R produces an error message. As this problem occurs only when consumption levels are estimated to be very low, the assumption was made that under such circumstances the AAF calculated with this set of parameters would also be 0. This method assumes that former drinkers are not at an elevated risk for the given disease.

In general, the scale (θ) and shape (κ) parameters of the gamma distributions are correlated and the covariance has to be taken into account when generating random samples of θ and κ. This difficulty is avoided considering the fact that κ is a constant in our case (it should be noted, however, that the constant is different for men and women). According to previous work by Rehm and colleagues:

where β is the coefficient linking the standard deviation to the mean. Therefore, κ is independent of region, age group and θ. In order to generate a random sample of κ, the variance is found using the delta method:

The generation of θ parameters is more difficult. Estimates of θ are different for each region, sex and age group since θ depends on the mean and variance of each gamma distribution. The generation of θ was performed in 2 steps: first, we generated a random sample of adult per capita consumption values from a normal distribution using the mean and standard deviation of this distribution, and second we generated a random sample of the prevalence of lifetime abstainers and former drinkers.

As the proportions of lifetime abstainers and former drinkers in each case follow binomial distributions, their variances, considering only sampling variation, can be expressed as follows:

The effective sample size of each survey used to estimate the proportion of lifetime abstainers and former drinkers was assumed to be 1000 (to reflect an average sample size for surveys of 6000 per population, assuming that the three age-sex categories have equal cell size). Using these values, it is possible to calculate the corresponding proportion of drinkers, the mean consumption per sex-age category and, finally, θ, which is then simply given by .

To account for the error of the final relative risk functions, N instances of each beta-coefficient were generated based on the covariance matrix. Each of these N relative risk functions obtained with one instance of each beta-coefficient was then assigned to one set of parameters defining the population (mean adult per capita consumption, proportion of abstainers and proportion of former drinkers). The relative risk functions were assumed to be the same for all regions and age groups.

As previously mentioned, each random set of lowest level parameters described above were then used to calculate an AAF value for a total of N AAFs for each region, sex, age group and disease. The variance of the N AAFs was used as the true variance of the AAF estimates.

### Main analysis, sensitivity analyses, and evaluation of the impact of each variable on the variance

As an example of this method, we calculated the AAFs for males aged 15 to 34 in the Asian regions; however, the above-described methods can also be used to calculate the AAFs for females. In addition, to demonstrate that partial AAFs and variances for these AAFs can be calculated for different consumption levels, we estimated the AAFs for cardiovascular diseases, ischemic stroke and diabetes for males aged 15 to 34 who are low consumers of alcohol (0 to 39.9 grams of alcohol per day), moderate consumers of alcohol (40 to 59.9 grams of alcohol per day) and heavy consumers of alcohol (60 to 150 grams of alcohol per day).

In order to accurately estimate the variance of an AAF we need to determine how many samples are required. Too few samples could lead to inaccurate results, while increasing the number of samples increases computing times and may require a larger amount of storage. Additionally, after a large number of iterations, the gain in accuracy is very small and does not provide new substantial information. Therefore, in order to determine the optimal number of random samples needed to calculate the variance of an AAF, a sensitivity analysis was performed. Since our samples are randomly generated, each set of samples is independent and allows us to collect a large amount of data relatively quickly. To decrease computation time, the code was adapted to generate 150 sets, each containing 1000 AAF estimates for each region (by sex and age and disease). The variance of each set of 1000 AAFs can then be averaged to estimate the variance of larger sets. By systematically increasing the number of sets used to calculate the average variance, we estimated the number of samples required for the variance to settle.

Next, we carried out an analysis to estimate the impact of each component on the final variance using the same sets of randomly generated variables, but in different arrangements. For the purposes of this analysis, only 1000 sets of lowest level parameters (see above for a definition) were generated.

To calculate the impact on the variance of each parameter, the AAFs were calculated for a set of parameters in which only the parameter tested was randomly generated while the other parameters were held constant. The variance obtained from the generated AAFs then represented the variance induced by the error of this single variable. Since the AAF function is non-linear, the variances obtained cannot simply be added together to obtain the total variance. To simplify the interpretation of the results, each contribution was normalized so that the sum equalled the total variance obtained as a result of the computation explained in the previous paragraphs. For the purpose of our analysis, the computations of the proportion of total variance explained by different variables were restricted to men in the five Asian regions defined above.

To compare in terms of dose response and magnitude the AAFs calculated using the new methodology by Rehm and colleagues to the method used in the 2004 CRA study [19], we calculated the partial AAFs for cardiovascular diseases and diabetes of multiple drinking categories for men in the five above defined Asian regions. The drinking categories were defined as

1) 0 to < 0.25 grams per day, 2) 0.25 to < 20 grams per day, 3) 20 to < 40 grams per day, and 4) 40+ grams per day. The relative risks used in the 2004 CRA study for cardiovascular diseases and diabetes were obtained from Gutjahr et al., [20], Reynolds et al., [21], Carrao et al., [22], and Corrao et al., [23].

### Considerations of computing time

As R is a single-core program, splitting up the code into different parts (for example, by sex and age) allows a user to take advantage of the multi-core architecture of modern central processing units. Additionally, when dealing with large data sets, R slows down considerably. The splitting of the program into different sub-programs by age, sex and region allows a user to reduce the size of the data sets, and therefore to speed up the computations.

## Results and Discussion

Per capita estimates by sex and region are shown in table 1, and prevalence of drinkers, former drinkers, and current drinkers are shown in table 2. We observed that on average men drink approximately 4.25 times more than women, and that regions with higher income levels and higher standards of living exhibited increased levels of alcohol consumption. The variance of per capita consumption is also much more important proportionally to the point estimate for countries with lower standards of living. The exhibited proportions of current drinkers bear out these conclusions.

**Table 1:** Per capita alcohol estimates (in grams per day)

Region		Men	S.E.
1	Asia, Pacific (high income)	13.51	1.57

2	Asia, Central	10.62	1.73

3	Asia, East	9.88	1.42

4	Asia, South	3.80	0.80

5	Asia, Southeast	5.21	0.79

**Table 2:** Prevalence of current drinkers, former drinkers and lifetime abstainers by region, and age

Region	Sex	lifetime abstainer	S.E.	former drinker	S.E.	current drinker	S.E.
1	M	0.05	0.01	0.07	0.01	0.87	0.01

2	M	0.23	0.01	0.13	0.01	0.64	0.02

3	M	0.13	0.01	0.15	0.01	0.72	0.01

4	M	0.73	0.01	0.11	0.01	0.17	0.01

5	M	0.56	0.02	0.17	0.01	0.27	0.01

Table 3 depicts the point estimates of each disease for the male population of the five described Asian regions, including their 95% CIs. When an AAF estimate was close to zero, the CIs also crossed zero, making it impossible to determine if the AAF was truly positive or negative. The use of 150 000 random samples provided us with enough precision to confidently estimate the CIs to two decimal places. In addition, the partial AAFs and their variances can be calculated for low consumers of alcohol (0 to 39.9 grams of alcohol per day), moderate consumers of alcohol (40 to 59.9 grams of alcohol per day), and heavy consumers of alcohol (60 to 150 grams of alcohol per day). Table 4 outlines the AAFs for cardiovascular diseases, ischemic stroke and diabetes by consumption amount.

**Table 3:** AAFs and 95% confidence intervals for the 5 Asian regions considering only the male population aged 15 - 34 years

		Asia, Pacific (High Income)			Asia, Central			Asia, East			Asia, South			Asia, Southeast	
**DISEASE**	**AAF**	**lower bound**	**upper bound**	**AAF**	**lower bound**	**upper bound**	**AAF**	**lower bound**	**upper bound**	**AAF**	**lower bound**	**upper bound**	**AAF**	**lower bound**	**upper bound**

Oral Cavity and Pharynx Cancer	43.00%	35.50%	50.40%	41.80%	32.60%	51.00%	25.70%	19.60%	31.80%	16.40%	10.20%	22.60%	22.70%	16.50%	28.90%

Oesophagus Cancer	25.90%	20.60%	31.30%	25.60%	18.70%	32.60%	14.90%	11.30%	18.50%	9.30%	5.80%	12.80%	13.50%	9.80%	17.20%

Colon Cancer	4.40%	1.60%	7.10%	4.70%	2.00%	7.40%	4.70%	2.70%	6.70%	2.70%	1.50%	4.00%	4.90%	2.80%	7.00%

Rectum Cancer	7.40%	5.10%	9.60%	7.40%	5.00%	9.80%	6.10%	4.20%	8.00%	3.40%	2.20%	4.70%	5.90%	3.90%	8.00%

Liver Cancer	13.40%	8.40%	18.40%	12.60%	7.50%	17.60%	9.10%	6.00%	12.10%	4.90%	2.90%	6.90%	8.10%	5.30%	10.90%

Larynx Cancer	27.70%	21.70%	33.60%	27.30%	19.70%	34.90%	15.80%	11.90%	19.80%	9.90%	6.10%	13.80%	14.30%	10.30%	18.30%

Coronary Heart Disease	-13.80%	-36.40%	8.80%	-5.40%	-24.10%	13.30%	-7.50%	-16.60%	1.60%	0.40%	-4.20%	5.10%	-0.40%	-6.80%	6.00%

Epilepsy	24.90%	17.80%	32.10%	24.60%	16.00%	33.30%	14.50%	10.30%	18.70%	8.90%	4.90%	13.00%	13.00%	8.80%	17.20%

Conduction Disorder and other Dysrhythmias	11.70%	7.30%	16.10%	11.40%	6.70%	16.10%	8.10%	5.40%	10.70%	4.60%	2.70%	6.40%	7.50%	4.90%	10.00%

Pancreatitis	19.90%	11.80%	28.00%	27.00%	12.80%	41.10%	7.80%	5.10%	10.50%	10.20%	3.40%	17.00%	11.00%	6.30%	15.70%

Lower Respiratory Infections	9.80%	2.50%	17.00%	9.60%	2.30%	16.90%	7.20%	3.40%	10.90%	4.10%	1.60%	6.50%	6.80%	3.50%	10.10%

Hemorrhagic Stroke - Morbidity	15.80%	12.20%	19.30%	15.70%	10.80%	20.70%	11.20%	4.70%	17.80%	6.70%	2.10%	11.30%	10.70%	2.90%	18.50%

Hemorrhagic Stroke - Mortality	14.20%	9.00%	19.50%	14.20%	8.00%	20.50%	10.60%	3.80%	17.40%	6.20%	1.50%	11.00%	10.10%	2.20%	18.10%

Ischemic Stroke - Morbidity	-4.70%	-11.80%	2.40%	1.80%	-4.40%	8.00%	-2.00%	-11.40%	7.40%	3.00%	-1.90%	7.80%	4.10%	-5.00%	13.10%

Ischemic Stroke	-4.30%	-11.30%	2.70%	2.30%	-3.80%	8.40%	-2.00%	-11.40%	7.40%	3.10%	-1.80%	8.00%	4.20%	-4.80%	13.20%

Tuberculosis	22.10%	12.50%	31.70%	23.20%	13.00%	33.40%	9.90%	5.30%	14.40%	8.40%	4.00%	12.80%	11.80%	6.50%	17.10%

Diabetes Mellitus	-5.30%	-17.80%	7.30%	-0.20%	-11.50%	11.20%	-2.50%	-10.30%	5.20%	1.40%	-2.80%	5.60%	1.50%	-5.40%	8.30%

Hypertension	17.50%	12.30%	22.70%	16.50%	10.30%	22.70%	8.40%	5.50%	11.40%	4.70%	2.20%	7.30%	6.80%	4.00%	9.50%

Liver Cirrhosis - Morbidity	34.20%	24.50%	43.90%	35.10%	22.60%	47.60%	19.80%	9.20%	30.50%	13.80%	4.60%	23.10%	18.80%	6.20%	31.40%

Liver Cirrhosis	57.00%	45.20%	68.90%	61.40%	45.80%	77.00%	32.10%	21.30%	43.00%	30.30%	15.10%	45.60%	34.00%	20.20%	47.80%

Computed using 150 000 sampled parameter sets

**Table 4:** AAFs and 95% confidence intervals for the 5 Asian regions based on consumption patterns considering only the male population aged 15 - 34 years

			Asia, Pacific (High Income)			Asia, Central			Asia, East			Asia, South			Asia, Southeast	
		
		AAF	lower bound	upper bound	AAF	lower bound	upper bound	AAF	lower bound	upper bound	AAF	lower bound	upper bound	AAF	lower bound	upper bound
Low consumption (0 to 39.9 grams of alcohol per day)	Coronary Heart Disease	-13.91%	-23.33%	-4.49%	-7.08%	-13.80%	-0.37%	-10.88%	-19.18%	-2.58%	-1.55%	-2.97%	-0.14%	-4.18%	-6.43%	-1.93%

	Conduction Disorder and other Dysrhythmias	5.58%	3.76%	7.40%	3.39%	1.95%	4.82%	3.86%	2.18%	5.55%	0.81%	0.38%	1.25%	1.92%	1.31%	2.53%

	Ischemic Stroke - Morbidity	-9.24%	-13.37%	-5.12%	-4.48%	-7.51%	-1.45%	-8.13%	-11.65%	-4.62%	-0.99%	-1.61%	-0.38%	-2.84%	-3.81%	-1.88%

	Ischemic Stroke - Mortality	-9.27%	-13.45%	-5.09%	-4.46%	-7.55%	-1.38%	-8.23%	-11.77%	-4.69%	-0.99%	-1.58%	-0.39%	-2.85%	-3.82%	-1.88%

	Diabetes Mellitus	-7.19%	-12.98%	-1.40%	-3.84%	-8.06%	0.38%	-5.49%	-10.65%	-0.32%	-0.87%	-1.76%	0.03%	-2.26%	-3.71%	-0.81%

	Hypertension	8.82%	6.98%	10.67%	5.44%	3.95%	6.94%	6.15%	4.43%	7.86%	1.33%	0.78%	1.88%	3.10%	2.37%	3.84%

																

Moderate consumption (40 to 60 grams of alcohol per day)	Coronary Heart Disease	-1.08%	-4.02%	1.85%	-0.88%	-3.29%	1.52%	-0.40%	-3.88%	3.09%	-0.21%	-1.20%	0.77%	-0.37%	-1.70%	0.96%

	Conduction Disorder and other Dysrhythmias	2.45%	1.41%	3.50%	2.05%	1.05%	3.05%	0.89%	-0.29%	2.07%	0.51%	0.16%	0.86%	0.85%	0.39%	1.31%

	Ischemic Stroke - Morbidity	0.55%	-0.02%	1.12%	0.47%	-0.01%	0.95%	0.19%	-0.48%	0.85%	0.12%	-0.07%	0.30%	0.19%	-0.06%	0.44%

	Ischemic Stroke - Mortality	0.69%	0.18%	1.20%	0.59%	0.14%	1.03%	0.24%	-0.36%	0.84%	0.14%	-0.03%	0.31%	0.24%	0.01%	0.46%

	Diabetes Mellitus	-0.45%	-2.12%	1.21%	-0.36%	-1.72%	1.00%	-0.17%	-2.16%	1.81%	-0.09%	-0.64%	0.47%	-0.15%	-0.91%	0.61%

	Hypertension	4.15%	2.71%	5.59%	3.48%	2.00%	4.96%	1.52%	-0.15%	3.18%	0.87%	0.38%	1.37%	1.45%	0.85%	2.06%

																

Heavy consumption (60+ grams of alcohol per day)	Coronary Heart Disease	0.22%	-3.00%	3.43%	0.60%	-2.47%	3.67%	-0.01%	-5.64%	5.61%	0.17%	-3.27%	3.60%	0.08%	-3.09%	3.25%

	Conduction Disorder and other Dysrhythmias	3.71%	2.11%	5.31%	5.21%	3.46%	6.96%	0.61%	-2.11%	3.33%	1.42%	-0.11%	2.96%	1.35%	-0.07%	2.77%

	Ischemic Stroke - Morbidity	1.91%	1.07%	2.75%	2.82%	1.91%	3.73%	0.29%	-1.19%	1.77%	0.76%	-0.09%	1.61%	0.69%	-0.07%	1.45%

	Ischemic Stroke - Mortality	2.17%	1.26%	3.07%	3.19%	2.17%	4.21%	0.33%	-1.25%	1.91%	0.86%	-0.07%	1.79%	0.78%	-0.02%	1.59%

	Diabetes Mellitus	1.34%	-0.81%	3.50%	2.31%	0.22%	4.40%	0.15%	-3.72%	4.02%	0.63%	-1.84%	3.11%	0.49%	-1.72%	2.70%

	Hypertension	6.70%	4.04%	9.36%	9.41%	6.40%	12.42%	1.11%	-3.31%	5.53%	2.66%	0.04%	5.28%	2.49%	0.15%	4.84%

### Impact on total variance of each parameter

**Figure 4:**
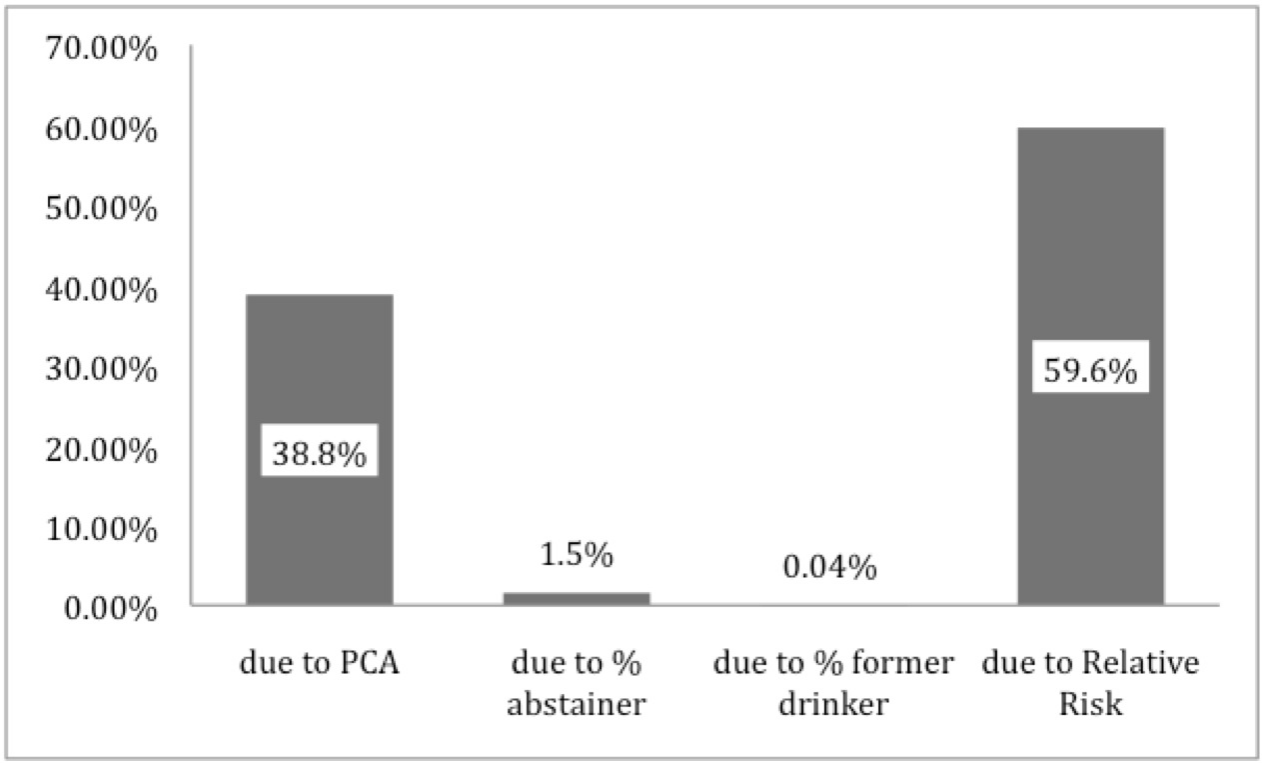
**Impact of parameters of AAF on final variance**.

As shown in Figure 4, the variance of the relative risk functions was, on average, the largest contributor to the variance of the AAFs. This is the case for disease categories such as ischemic heart disease and lower respiratory infections, where the variance of the betas of the relative risk function for these diseases is large [5]. For oral cavity cancer, oesophageal cancer, larynx cancer, pancreatitis, tuberculosis, liver cirrhosis and hemorrhagic stroke, adult per capita consumption was the largest contributor to the variance observed in the AAFs. We can speculate that either more research has been undertaken concerning those diseases leading to more precise risk functions, or that simple relationships can be more accurately estimated with fewer errors.

### Examples of changes in variance as the sample size increases

**Figure 5:**
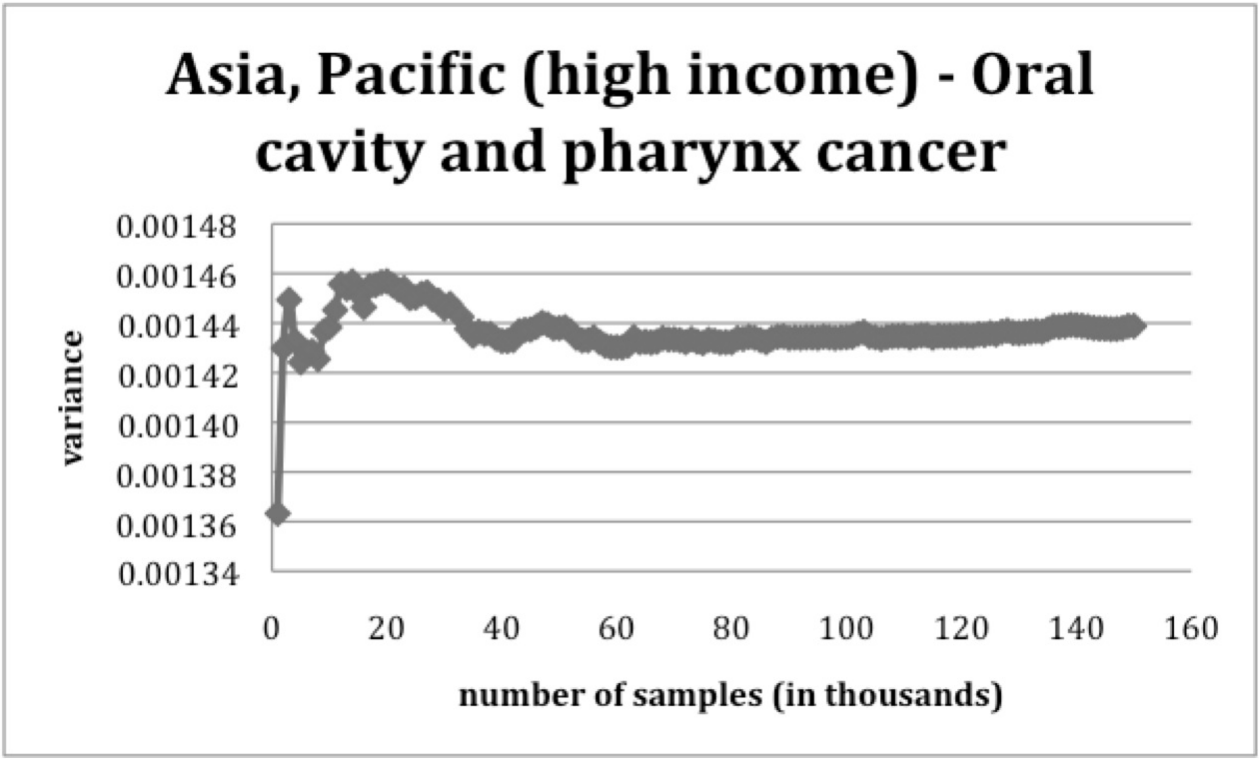
**Change of the estimated variance of oral cavity and pharynx cancer in Asia, Pacific (high income) for the male population aged between 15 and 34 years as the sample size increases**.

**Figure 6:**
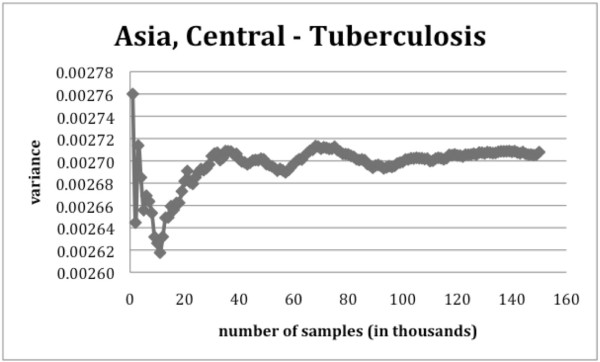
**Change of the estimated variance of tuberculosis in Asia, Central for the male population aged between 15 and 34 years as the sample size increases**.

Figures 5 and 6 illustrate the change in the estimated variance when more samples are added to the analysis. When looking at the convergence of the variance, in the case of oral cavity and pharynx cancer, approximately 60 000 to 70 000 random samples are required to accurately estimate the variance of the AAFs. For the case of tuberculosis in Asia Central, we found that the variance converged only after 100 000 random samples were used. Therefore, in order to insure the convergence of each variance estimate considering a small safety margin, around 150 000 sample points are needed. It should be noted, however, that if the CIs are to be determined with a maximum error of ± 1 on the second decimal, the precision of the variance only needs to be greater than 2.6e-5 which is usually achieved after as few as 40 000 samples.

### Comparison of AAFs using new and old methodologies

Table 5 outlines the new methodology of calculating the AAF by Rehm and colleagues [4] and the methodology used in the 2004 CRA study. The AAFs for the new methodology are very close to the AAFs estimated using the old methodology for the same drinking groups; however, the inclusion of former drinkers in the new methodology increases the total AAF for each region. Also, it should be stated that prevalence of consumption in this sensitivity analysis was based on the gamma distribution for both methodologies (once used continuous and once categorical), whereas the original 2004 CRA study used a different form of up estimation [13].

**Table 5:** AAFs for the 5 Asian regions based on methods used in the 2004 CRA study and methods used in the 2005 CRA study for males aged 15 - 34 years

		Asia, Pacific (High Income)		Asia, Central		Asia, East		Asia, South		Asia, Southeast	
		
		New Method	Old Method	New Method	Old Method	New Method	Old Method	New Method	Old Method	New Method	Old Method
Abstainers, Former drinkers or very light drinkers	Coronary Heart Disease	0.71%	0.00%	1.63%	0.00%	2.93%	0.00%	1.95%	0.00%	3.68%	0.00%

	Ischemic Stroke - Morbidity	1.27%	0.00%	2.61%	0.00%	4.68%	0.00%	3.05%	0.00%	5.71%	0.00%

	Ischemic Stroke - Mortality	1.26%	0.00%	2.61%	0.00%	4.67%	0.00%	3.05%	0.00%	5.71%	0.00%

	Diabetes Mellitus	0.66%	0.00%	1.43%	0.00%	2.57%	0.00%	1.68%	0.00%	3.19%	0.00%

	Hypertension	-0.09%	0.00%	-0.04%	0.00%	-0.10%	0.00%	-0.01%	0.00%	-0.03%	0.00%

											

Drinking Category I (0.25 to < 40 grams of alcohol per day)	Coronary Heart Disease	-18.18%	-20.30%	-8.74%	-9.52%	-15.26%	-17.50%	-1.89%	-2.04%	-5.30%	-5.84%

	Ischemic Stroke - Morbidity	-13.22%	-8.46%	-6.08%	-4.03%	-12.36%	-7.90%	-1.33%	-0.90%	-3.96%	-2.61%

	Ischemic Stroke - Mortality	-13.25%	-8.46%	-6.07%	-4.03%	-12.46%	-7.90%	-1.32%	-0.90%	-3.96%	-2.61%

	Diabetes Mellitus	-11.05%	-4.19%	-5.44%	-1.91%	-9.55%	-4.34%	-1.20%	-0.43%	-3.37%	-1.32%

	Hypertension	6.03%	7.47%	4.11%	4.35%	2.93%	5.61%	1.01%	1.04%	2.10%	2.59%

											

Drinking Category II (40 to < 60 grams of alcohol per day)	Coronary Heart Disease	-1.08%	-4.81%	-0.88%	-2.63%	-0.40%	-4.18%	-0.21%	-0.60%	-0.37%	-1.53%

	Ischemic Stroke - Morbidity	0.55%	-2.30%	0.47%	-0.64%	0.19%	-3.27%	0.12%	-0.13%	0.19%	-0.72%

	Ischemic Stroke - Mortality	0.69%	-2.30%	0.59%	-0.64%	0.24%	-3.27%	0.14%	-0.13%	0.24%	-0.72%

	Diabetes Mellitus	-0.45%	-7.08%	-0.36%	-4.42%	-0.17%	-4.98%	-0.09%	-1.01%	-0.15%	-2.25%

	Hypertension	4.15%	0.86%	3.48%	1.89%	1.52%	-2.08%	0.87%	0.49%	1.45%	0.35%

											

Drinking Category III (60+ grams of alcohol per day)	Coronary Heart Disease	0.22%	-3.38%	0.60%	-1.49%	-0.01%	-3.66%	0.17%	-0.33%	0.08%	-1.07%

	Ischemic Stroke - Morbidity	1.91%	-2.13%	2.82%	0.06%	0.29%	-3.43%	0.76%	0.07%	0.69%	-0.64%

	Ischemic Stroke - Mortality	2.17%	-2.13%	3.19%	0.06%	0.33%	-3.43%	0.86%	0.07%	0.78%	-0.64%

	Diabetes Mellitus	1.34%	-5.20%	2.31%	-3.79%	0.15%	-3.98%	0.63%	-0.91%	0.49%	-1.68%

	Hypertension	6.70%	3.95%	9.41%	7.53%	1.11%	-2.25%	2.66%	2.11%	2.49%	1.53%

## Conclusions

In this paper we have presented a method to estimate uncertainty around AAFs and illustrated our results using data for men aged 15 to 34 years in several Asian regions. The use of 60 000 to 70 000 Monte Carlo samples yields stable variance estimates in most cases, but we propose the use of 150 000 samples to ensure stable CIs. Uncertainties about risk relations and about total per capita consumption were identified as the main contributors to variances of the AAFs for men aged 15 to 34 years in the five Asian regions. These variances indicate that for some disease categories the dose-response curve from alcohol has not been sufficiently researched. The observed large variances may result from an insufficient number of underlying articles describing dose-response or from the non-linear nature of dose-response relationships. As some of the non-linear nature may be caused by other dimensions of alcohol consumption (for example, irregular heavy drinking occasions in the case of ischemic diseases) [24,25], it will not be enough to just conduct more epidemiological studies into the impact of average volume of alcohol consumption on the incidence of diseases (for an overview see [5]). Instead, other relevant dimensions of alcohol consumption, which could play a role in confounding the average volume of alcohol consumption, should be included in the design of cohort studies, and then should be statistically controlled for by using, for example, meta-regression techniques [26].

One limitation of our approach was the use of adjusted relative risks in determining AAFs. The relative risk formulas we used were developed for risks only adjusted for age (see [8,9,27]). Two arguments can be made to justify the use of these formulas. Firstly, in risk analyses, such as the CRA for the GBD Studies [28], almost all of the underlying studies for the different risk factors report only adjusted risks. Relying on unadjusted risks would severely bias the estimated risk functions as only a small proportion of generally older studies could be included. Secondly, for alcohol in particular, most of the analyses show no marked differences after adjustment for the usual risk factors tested (see [5], and the meta-analyses cited there). The need for adjustment to the relative risks may change when other dimensions of alcohol consumption, such as irregular heavy drinking occasions, are considered (see above).

Another limitation of the new methodology is the nature of the relative risks that are used in the CRA study. As there is likely to be undercoverage of alcohol consumption in the medical epidemiological studies upon which the relative risks are based, modelling 100% of adult per capita consumption will lead to biased results. Accordingly, as coverage of alcohol consumption in these studies is likely greater than 70% [10], we modelled alcohol consumption as 80% of adult per capita consumption. This adjustment leads to lower estimates of alcohol-attributable health harms [10]. Additionally, we modelled average daily alcohol consumption from 0 to 150 grams a day, using 150 grams as a maximum level. In very rare cases people may drink more than 150 grams per day; however, it is unlikely that this level of consumption would be maintained over an extended period of time [29]. An upper limit of alcohol consumption in grams per day may lead to an underestimation of the effects of alcohol in terms of total harms, especially where alcohol at low doses has a positive effect and at high doses has a negative effect, such as with cardiovascular diseases, ischemic stroke and diabetes. Such instances are limited, however, as the risk ratios used to model the effects of alcohol were fractional polynomials allowing us to accurately characterize curvilinear risk relationships. Additionally, alcohol starts to have a negative effect well below a consumption level of 150 grams per day and, thus, limiting our consumption models to 150 grams per day does not have a substantial effect on the AAFs. Furthermore, as the upper limit of sustainable alcohol consumption probably differs depending on the sex of the drinker, more research is needed to define these limits.

Our new methodology is capable of being adjusted to take into account different parameters of alcohol consumption [10]. For example, this method can easily be modified for future research that focuses on the effects of specific alcohol consumption patterns on the burden of disease. In summary, future iterations of the CRA, or similar studies, should include CIs, as our methodology offers a feasible way to estimate the uncertainty of attributable fractions for all burdens of disease.

## Competing interests

The authors declare that they have no competing interests.

## Authors' contributions

Gerrit G helped to develop the mathematical model, the code and contributed to the writing. KS helped analysing the data and writing the article. HF helped optimising the code. TK helped in developing the mathematical model. JR and Gerhard G conceptualised the overall article and contributed to the writing. All authors read and approved the final version.

## Pre-publication history

The pre-publication history for this paper can be accessed here:

http://www.biomedcentral.com/1471-2288/11/48/prepub

## Supplementary Material

Additional file 1 **Example of R - code for simulations**. Click here for file
