# Supplementary material for: Estimating uncertainty of alcohol-attributable fractions for infectious and chronic diseases
Source: BMC Med Res Methodol. 2011 Apr 17;11:48. doi: 10.1186/1471-2288-11-48 (PMC3088897; doi:10.1186/1471-2288-11-48)
Supplement: Additional file 1 — Example of R - code for simulations. [file 1471-2288-11-48-S1.DOCX]

**Appendix: R code used for our main analysis**

############# Definition of Relative Risk Functions as well as the Variances and Covariances of the parameters ###########

############# In order to use them in a loop, we need a constant type in which we will store the parameters ###########

############# As there are 4 different functions: sqrt(x), x, x^2 and x^3 we will use 4 beta parameters for ###########

############# each RR function. If the function only has a few of there terms, the other coefficients will ###########

############# simply equal zero. The cross correlation terms will also be voided. Hence each function will have ###########

############# its 4x4 matrix including all the needed information. ###########

############# The beta coefficients will represent respectively the coeffients for sqrt(x), x, x^2 and x^3 ###########

############# Note: the Relative Risk functions have sometimes been changed at the extremities, therefore, in ###########

############# order to be able to simulate our RR functions with different beta parameters, the easiest is to ###########

############# change the functions parameters compared to the previous ones. Namely, instead of being only a ###########

############# function of x, the relative risk functions will be functions of x and the different betas. ###########

############# To calculate the Confidence Intervals, we will therefore first generate the beta coefficients ###########

############# and then plug them into our Relative Risk function that we can use to calculate the AAFs. ###########

############# The values of the relative risks for former drinkers are given as their logarithmic values and ###########

############# the variance applies to these logarithmic parameters. Therefore, for the simulations, we'll have ###########

############# To simulate n logarithmic values and take the exponential ###########

####### Oral Cavity and Pharynx Cancer #######

####### Oral Cavity and Pharynx Cancer #######

#### male ####

RRoralma =

oralmabetas =

oralmacovar =

lnRRoralmaform=

lnRRoralmaformvar=

oralcancermale = list ("Oral Cavity and Pharynx Cancer - MEN", RRoralma, oralmabetas, oralmacovar,lnRRoralmaform,lnRRoralmaformvar)

#### female ####

RRoralfe=

oralfebetas =

oralfecovar =

lnRRoralfeform =

lnRRoralfeformvar =

oralcancerfemale = list ("Oral Cavity and Pharynx Cancer - WOMEN", RRoralfe, oralfebetas, oralfecovar,lnRRoralfeform,lnRRoralfeformvar)

####### Oral Oesophagus Cancer ###########

####### Oral Oesophagus Cancer ###########

##### male ####

RRoesoma =

oesomabetas =

oesomacovar =

lnRRoesomaformvar=

oesophaguscancermale = list("Oesophagus Cancer - MEN", RRoesoma, oesomabetas, oesomacovar,lnRRoesomaform,lnRRoesomaformvar)

#### female ####

RRoesofe =

oesofebetas =

oesofecovar =

lnRRoesofeform=

lnRRoesofeformvar=

oesophaguscancerfemale = list("Oesophagus Cancer - WOMEN", RRoesofe, oesofebetas, oesofecovar,lnRRoesofeform,lnRRoesofeformvar)

######## Colon Cancer ########

######## Colon Cancer ########

#### male ####

RRcoloma =

colomabetas =

colomacovar =

lnRRcolomaform=

lnRRcolomaformvar=

coloncancermale = list ("Colon Cancer - MEN", RRcoloma, colomabetas, colomacovar,lnRRcolomaform,lnRRcolomaformvar)

#### female ####

RRcolofe =

colofebetas =

colofecovar =

lnRRcolofeform =

lnRRcolofeformvar =

coloncancerfemale = list("Colon Cancer - WOMEN", RRcolofe, colofebetas, colofecovar,lnRRcolofeform,lnRRcolofeformvar)

######## Rectum Cancer #########

######## Rectum Cancer #########

#### male ####

RRrectma=

rectmabetas =

rectmacovar =

lnRRrectmaform =

lnRRrectmaformvar =

rectumcancermale = list("Rectum Cancer - MEN", RRrectma, rectmabetas, rectmacovar,lnRRrectmaform,lnRRrectmaformvar)

#### female ####

RRrectfe=

rectfebetas =

rectfecovar =

lnRRrectfeform =

lnRRrectfeformvar =

rectumcancerfemale = list("Rectum Cancer - WOMEN", RRrectfe, rectfebetas, rectfecovar,lnRRrectfeform,lnRRrectfeformvar)

######## Liver Cancer ########

######## Liver Cancer ########

#### male ####

RRlivema =

livemabetas =

livemacovar =

lnRRlivemaform =

lnRRlivemaformvar =

livercancermale = list("Liver Cancer - MEN", RRlivema, livemabetas, livemacovar,lnRRlivemaform,lnRRlivemaformvar)

#### female ####

RRlivefe =

livefebetas =

livefecovar =

lnRRlivefeform =

lnRRlivefeformvar =

livercancerfemale = list("Liver Cancer - WOMEN", RRlivefe, livefebetas, livefecovar,lnRRlivefeform,lnRRlivefeformvar)

######## Larynx Cancer #########

######## Larynx Cancer #########

#### male ####

RRlaryma =

larymabetas =

larymacovar =

lnRRlarymaform =

lnRRlarymaformvar =

larynxcancermale = list("Larynx Cancer - MEN", RRlaryma, larymabetas, larymacovar,lnRRlarymaform,lnRRlarymaformvar)

#### female ####

RRlaryfe =

laryfebetas =

laryfecovar =

lnRRlaryfeform =

lnRRlaryfeformvar =

larynxcancerfemale = list("Larynx Cancer - WOMEN", RRlaryfe, laryfebetas, laryfecovar,lnRRlaryfeform,lnRRlaryfeformvar)

####### Breast Cancer #######

####### Breast Cancer #######

#### male ####

###∞∞∞ not applicable ∞∞∞###

#### female ####

RRbreafe =

breafebetas =

breafecovar =

lnRRbreafeform =

lnRRbreafeformvar =

breastcancerfemale = list("Breast Cancer - WOMEN", RRbreafe, breafebetas, breafecovar,lnRRbreafeform,lnRRbreafeformvar)

####### Coronary Heart Disease #######

####### Coronary Heart Disease #######

#### male ####

RRcoroma =

coromabetas =

coromacovar =

lnRRcoromaform =

lnRRcoromaformvar =

coronaryheartdiseasemale = list ("Coronary Heart Disease - MEN", RRcoroma, coromabetas, coromacovar,lnRRcoromaform,lnRRcoromaformvar)

#### female ####

RRcorofe = RRcoroma=

corofebetas =

corofecovar =

lnRRcorofeform =

lnRRcorofeformvar =

coronaryheartdiseasefemale = list("Coronary Heart Disease - WOMEN", RRcorofe, corofebetas, corofecovar,lnRRcorofeform,lnRRcorofeformvar)

####### Epilepsy #######

####### Epilepsy #######

#### male ####

RRepilma =

epilmabetas =

epilmacovar =

lnRRepilmaform =

lnRRepilmaformvar =

epilepsymale = list("Epilepsy - MEN", RRepilma, epilmabetas, epilmacovar,lnRRepilmaform,lnRRepilmaformvar)

#### female ####

RRepilfe =

epilfebetas =

epilfecovar =

lnRRepilfeform =

lnRRepilfeformvar =

epilepsyfemale = list("Epilepsy - WOMEN", RRepilfe, epilfebetas, epilfecovar,lnRRepilfeform,lnRRepilfeformvar)

####### Conduction Disorders and other Dysrythmias #######

####### Conduction Disorders and other Dysrythmias #######

#### male ####

RRcondma=

condmabetas =

condmacovar =

lnRRcondmaform =

lnRRcondmaformvar =

conductiondisordermale = list("Conduction Disorder and other Dysrythmias - MEN", RRcondma, condmabetas, condmacovar,lnRRcondmaform,lnRRcondmaformvar)

#### female ####

RRcondfe=

condfebetas =

condfecovar =

lnRRcondfeform =

lnRRcondfeformvar =

conductiondisorderfemale = list("Conduction Disorder and other Dysrythmias - WOMEN", RRcondfe, condfebetas, condfecovar,lnRRcondfeform,lnRRcondfeformvar)

####### Pancreatitis #######

#### male ####

RRpancma =

pancmabetas =

pancmacovar =

lnRRpancmaform =

lnRRpancmaformvar =

pancreatitismale = list("Pancreatitis - MEN", RRpancma, pancmabetas, pancmacovar,lnRRpancmaform,lnRRpancmaformvar)

#### female ####

RRpancfe =

pancfebetas =

pancfecovar =

lnRRpancfeform =

lnRRpancfeformvar =

pancreatitisfemale = list("Pancreatitis - WOMEN", RRpancfe, pancfebetas, pancfecovar,lnRRpancfeform,lnRRpancfeformvar)

####### Lower Respiratory Infections ########

####### Lower Respiratory Infections ########

#### male ####

RRlowema =

lowemabetas =

lowemacovar =

lnRRlowemaform =

lnRRlowemaformvar =

lowerrespmale = list("Lower Respiratory Infections - MEN", RRlowema, lowemabetas, lowemacovar,lnRRlowemaform,lnRRlowemaformvar)

#### female ####

RRlowefe =

lowefebetas =

lowefecovar =

lnRRlowefeform =

lnRRlowefeformvar =

lowerrespfemale = list("Lower Respiratory Infections - WOMEN", RRlowefe, lowefebetas, lowefecovar,lnRRlowefeform,lnRRlowefeformvar)

####### Hemorrhagic Stroke - Morbidity ##########

####### Hemorrhagic Stroke - Morbidity ##########

#### male ####

RRmorbhemoma =

morbhemomabetas =

morbhemomacovar =

lnRRmorbhemomaform =

lnRRmorbhemomaformvar =

hemorrhagicstrokemorbiditymale = list ("Hemorrhagic Stroke - Morbidity - MEN", RRmorbhemoma, morbhemomabetas, morbhemomacovar,lnRRmorbhemomaform,lnRRmorbhemomaformvar)

#### female ####

RRmorbhemofe=

morbhemofebetas =

morbhemofecovar =

lnRRmorbhemofeform =

lnRRmorbhemofeformvar =

hemorrhagicstrokemorbidityfemale = list("Hemorrhagic Stroke - Morbidity - WOMEN", RRmorbhemofe, morbhemofebetas, morbhemofecovar,lnRRmorbhemofeform,lnRRmorbhemofeformvar)

####### Hemorrhagic Stroke - Mortality #########

####### Hemorrhagic Stroke - Mortality #########

#### male ####

RRhemoma =

hemomabetas =

hemomacovar =

lnRRhemomaform =

lnRRhemomaformvar =

hemorrhagicstrokemale = list("Hemorrhagic Stroke - Mortality - MEN", RRhemoma, hemomabetas, hemomacovar,lnRRhemomaform,lnRRhemomaformvar)

#### female ####

RRhemofe =

hemofebetas =

hemofecovar =

lnRRhemofeform =

lnRRhemofeformvar =

hemorrhagicstrokefemale = list("Hemorrhagic Stroke - Mortality - WOMEN", RRhemofe, hemofebetas, hemofecovar,lnRRhemofeform,lnRRhemofeformvar)

###### Ischemic Stroke - Morbidity #######

###### Ischemic Stroke - Morbidity #######

#### male ####

##### remark: this function is somehow special as it includes a sqrt(x)*ln(x) function. Therefore, the beta2 coefficient will represent

##### this factor and not the coefficient for x^1.

RRmorbischma=

morbischmabetas =

morbischmacovar =

lnRRmorbischmaform =

lnRRmorbischmaformvar =

ischemicstrokemorbiditymale = list("Ischemic Stroke - Morbidity - MEN", RRmorbischma, morbischmabetas, morbischmacovar,lnRRmorbischmaform, lnRRmorbischmaformvar)

#### female ####

RRmorbischfe=

morbischfebetas =

morbischfecovar =

lnRRmorbischfeform =

lnRRmorbischfeformvar =

ischemicstrokemorbidityfemale = list("Ischemic Stroke - Morbidity - WOMEN", RRmorbischfe, morbischfebetas, morbischfecovar,lnRRmorbischfeform,lnRRmorbischfeformvar)

####### Ischemic Stroke - Mortality #######

####### Ischemic Stroke - Mortality #######

#### male ####

RRischma =

ischmabetas =

ischmacovar =

lnRRischmaform =

lnRRischmaformvar =

ischemicstrokemale = list("Ischemic Stroke - MEN", RRischma, ischmabetas, ischmacovar,lnRRischmaform,lnRRischmaformvar)

#### female ####

RRischfe=

ischfebetas =

ischfecovar =

lnRRischfeform =

lnRRischfeformvar =

ischemicstrokefemale = list ("Ischemic Stroke - WOMEN", RRischfe, ischfebetas, ischfecovar,lnRRischfeform,lnRRischfeformvar)

####### Tuberculosis #######

####### Tuberculosis #######

#### remark: this is a piecewise constant function ####

#### male ####

RRtubema =

tubemabetas =

tubemacovar =

lnRRtubemaform =

lnRRtubemaformvar =

tuberculosismale = list("Tuberculosis - MEN", RRtubema, tubemabetas, tubemacovar,lnRRtubemaform,lnRRtubemaformvar)

#### female ####

RRtubefe =

tubefebetas =

tubefecovar =

lnRRtubefeform =

lnRRtubefeformvar =

tuberculosisfemale = list ("Tuberculosis - WOMEN", RRtubefe, tubefebetas, tubefecovar,lnRRtubefeform,lnRRtubefeformvar)

####### Diabetes Mellitus #######

####### Diabetes Mellitus #######

#### male ####

RRdiabma =

diabmabetas =

diabmacovar =

lnRRdiabmaform =

lnRRdiabmaformvar =

diabetesmale = list ("Diabetes Mellitus - MEN", RRdiabma, diabmabetas, diabmacovar,lnRRdiabmaform,lnRRdiabmaformvar)

#### female ####

RRdiabfe =

diabfebetas =

diabfecovar =

lnRRdiabfeform =

lnRRdiabfeformvar =

diabetesfemale = list ("Diabetes Mellitus - WOMEN", RRdiabfe, diabfebetas, diabfecovar,lnRRdiabfeform,lnRRdiabfeformvar)

####### Hypertension #######

####### Hypertension #######

#### male ####

RRhypema=

hypemabetas =

hypemacovar =

lnRRhypemaform =

lnRRhypemaformvar =

hypertensionmale = list("Hypertension - MEN", RRhypema, hypemabetas, hypemacovar,lnRRhypemaform, lnRRhypemaformvar)

#### female ####

RRhypefe=

hypefebetas =

hypefecovar =

lnRRhypefeform =

lnRRhypefeformvar =

hypertensionfemale = list("Hypertension - WOMEN", RRhypefe, hypefebetas, hypefecovar,lnRRhypefeform,lnRRhypefeformvar)

####### Liver Cirrhosis - Morbidity #######

####### Liver Cirrhosis - Morbidity #######

#### male ####

RRmorbcirrma=

morbcirrmabetas =

morbcirrmacovar =

lnRRmorbcirrmaform =

lnRRmorbcirrmaformvar =

livercirrhosismorbiditymale = list ("Liver Cirrhosis - Morbidity - MEN", RRmorbcirrma, morbcirrmabetas, morbcirrmacovar,lnRRmorbcirrmaform,lnRRmorbcirrmaformvar)

#### female ####

RRmorbcirrfe =

morbcirrfebetas =

morbcirrfecovar =

lnRRmorbcirrfeform =

lnRRmorbcirrfeformvar =

livercirrhosismorbidityfemale = list("Liver Cirrhosis - Morbidity - WOMEN", RRmorbcirrfe, morbcirrfebetas, morbcirrfecovar,lnRRmorbcirrfeform,lnRRmorbcirrfeformvar)

####### Liver Cirrhosis - Mortality #######

####### Liver Cirrhosis - Mortality #######

#### male ####

RRcirrma=

cirrmabetas =

cirrmacovar =

lnRRcirrmaform =

lnRRcirrmaformva r=

livercirrhosismale = list ("Liver Cirrhosis - MEN", RRcirrma, cirrmabetas, cirrmacovar,lnRRcirrmaform,lnRRcirrmaformvar)

#### female ####

RRcirrfe=

cirrfebetas =

cirrfecovar =

lnRRcirrfeform =

lnRRcirrfeformvar =

livercirrhosisfemale = list ("Liver Cirrhosis - WOMEN", RRcirrfe, cirrfebetas, cirrfecovar,lnRRcirrfeform,lnRRcirrfeformvar)

################ Creating a list of all the diseases ####################

relativeriskmale = list(oralcancermale, oesophaguscancermale, coloncancermale, rectumcancermale, livercancermale, larynxcancermale,coronaryheartdiseasemale,epilepsymale,conductiondisordermale,pancreatitismale, lowerrespmale,hemorrhagicstrokemorbiditymale, hemorrhagicstrokemale,ischemicstrokemorbiditymale, ischemicstrokemale,tuberculosismale, diabetesmale,hypertensionmale, livercirrhosismorbiditymale, livercirrhosismale)

relativeriskfemale = list(oralcancerfemale, oesophaguscancerfemale, coloncancerfemale, rectumcancerfemale,livercancerfemale, larynxcancerfemale,breastcancerfemale, coronaryheartdiseasefemale,epilepsyfemale,conductiondisorderfemale,pancreatitisfemale, lowerrespfemale,hemorrhagicstrokemorbidityfemale,hemorrhagicstrokefemale,ischemicstrokemorbidityfemale,ischemicstrokefemale,tuberculosisfemale, diabetesfemale, hypertensionfemale, livercirrhosismorbidityfemale, livercirrhosisfemale)

#################∞∞∞∞∞∞∞∞∞∞∞∞∞∞∞∞∞∞∞∞∞∞∞∞∞∞∞∞∞∞∞∞∞∞∞∞∞∞ INPUT FILE ∞∞∞∞∞∞∞∞∞∞∞∞∞∞∞∞∞∞∞∞∞∞∞∞∞∞∞∞∞∞∞∞∞∞∞∞∞∞∞#############################

#################∞∞∞∞∞∞∞∞∞∞∞∞∞∞∞∞∞∞∞∞∞∞∞∞∞∞∞∞∞∞∞∞∞∞∞∞∞∞ INPUT FILE ∞∞∞∞∞∞∞∞∞∞∞∞∞∞∞∞∞∞∞∞∞∞∞∞∞∞∞∞∞∞∞∞∞∞∞∞∞∞∞#############################

#################∞∞∞∞∞∞∞∞∞∞∞∞∞∞∞∞∞∞∞∞∞∞∞∞∞∞∞∞∞∞∞∞∞∞∞∞∞∞ INPUT FILE ∞∞∞∞∞∞∞∞∞∞∞∞∞∞∞∞∞∞∞∞∞∞∞∞∞∞∞∞∞∞∞∞∞∞∞∞∞∞∞#############################

#################∞∞∞∞∞∞∞∞∞∞∞∞∞∞∞∞∞∞∞∞∞∞∞∞∞∞∞∞∞∞∞∞∞∞∞∞∞∞ INPUT FILE ∞∞∞∞∞∞∞∞∞∞∞∞∞∞∞∞∞∞∞∞∞∞∞∞∞∞∞∞∞∞∞∞∞∞∞∞∞∞∞#############################

############ Reading in and Formatting the input file #############

############ Reading in and Formatting the input file #############

######## The inputs are as follows:

######## REGION - SEX - AGE CATEGORY - %LIFETIME ABSTAINERS - %FORMER DRINKERS - %DRINKERS - POPULATION WEIGHT - RELATIVE COEFFICIENT - PCA (by sex) - SE of PCA

##### The AGE_CATEGORY variables have to be numbers. This is required in order for the program to recognise the different categories automatically. This will enable

##### us to change the number of age categories at any time

##### ATTENTION: PCA HAS TO BE GIVEN IN LITRES/YEAR!!! IT IS ALSO A REDUNDANT NUMBER INSIDE EACH REGION AS IT REPRESENTS THE TOTAL PCA OF THE REGION IN QUESTON.

##### IT IS KEPT REDUNDANT ONLY FOR THE SIMPLICITY OF THE PROGRAM DESIGN.

##### IN ADDITION TO THAT, THE PROGRAM WILL TAKE THE 80% OF THE INPUT PCA

input=read.delim("AAFinputfile.txt", header=F, colClasses=c("character","numeric","numeric","numeric","numeric","numeric","numeric","numeric","numeric","numeric"))

colnames(input)=c("REGION","SEX","AGE_CATEGORY","LIFETIME_ABSTAINERS","FORMER_DRINKERS","DRINKERS","POPULATION", "RELATIVE_COEFFICIENT","PCA","VAR_PCA")

inputm = input[input$SEX==1,] ####### male dataset

inputmage=inputm[inputm$AGE_CATEGORY==1,]

prop_abs_male = inputmage$LIFETIME_ABSTAINERS

prop_form_male = inputmage$FORMER_DRINKERS

#### the list of regions will be used to compute the mean values ####

#### indeed, each region has 6 different entries (2x3 for sex ####

#### and age group). We therefore need a non redundant list of ####

#### the regions. ####

agecategories_male=unique(inputmage$AGE_CATEGORY)

#### male ####

regions_male = unique(inputmage$REGION)

######## Determining the MEAN values #########

######## Determining the MEAN values #########

######## Determining the MEAN values #########

#### the generation of the mean values will need the use of a function ####

#### which we will be able to re-use for the CI by generating random ####

#### inputs and returning the output. ####

#### p1,p2 and p3 are the size of populations, a,b and c the relative coefficients; ####

#### the proportion of drinkers is directly related to the proportions of abstainers and former drinkers. the rest is straightforward ####

#### THE OUTPUT IS A VECTOR OF THE X MEAN VALUES CORRESPONDING TO THE X AGE GROUPS. ####

compute_mu=function (p1,p2,p3,a,b,c,prop_abs1,prop_abs2,prop_abs3,prop_form1,prop_form2,prop_form3, pca)

{

#### calculating the mean consumption of the drinking population ####

drk1=p1*(1-prop_abs1-prop_form1)

drk2=p2*(1-prop_abs2-prop_form2)

drk3=p3*(1-prop_abs3-prop_form3)

pca_drinker = pca*(p1+p2+p3)/(drk1+drk2+drk3)

mu1=pca_drinker*(drk1+drk2+drk3)/(drk1+b/a*drk2+c/a*drk3)

mu2=b/a*mu1

mu3=c/a*mu1

mu=c(mu1,mu2,mu3)

return(mu)

}

######## male population #######

n_agecategories_male = length(agecategories_male)

# The idea is to create a vector with NAs at the beginning and then fill it. Then you don't have to use the combine-command so often which slows things down.

mean_male=rep(NA, length = length(regions_male)*n_agecategories_male)

for (i in 1:length(regions_male))

{

#### extracting the required information for each region ####

info_male_region = inputm[inputm$REGION==regions_male[i],]

#### calculating the mean values for each group

population = info_male_region$POPULATION

coeffs=info_male_region$RELATIVE_COEFFICIENT

prop_abs=info_male_region$LIFETIME_ABSTAINERS

prop_form = info_male_region$FORMER_DRINKERS

pcalitresperyear=info_male_region$PCA[1] #### the three values for pca are redundant (one value only for the whole region), the choice to take the first one is arbitrary of course

pcagramsperday=pcalitresperyear*1000*0.789*0.8/365 #### This is to calculate the 80% pca

pca=pcagramsperday

mean_region = compute_mu(population[1],population[2],population[3],coeffs[1],coeffs[2],coeffs[3],prop_abs[1],prop_abs[2],prop_abs[3],prop_form[1],prop_form[2],prop_form[3],pca)

mean_male[i] = mean_region[1]

}

sd_male = 1.171*mean_male

k_male = mean_male^2/sd_male^2

theta_male = sd_male^2/mean_male

nmale = length (k_male) ##### number of different distributions corresponding to male populations

################### Calculation of AAF for male population ########################

################### Calculation of AAF for male population ########################

################### Calculation of AAF for male population ########################

#### male population ####

AAFinfolistmale = data.frame(matrix(NA, nrow=nmale*length(relativeriskmale), ncol=5))

names(AAFinfolistmale) = c("REGION","SEX","AGE_CATEGORY","DISEASE","AAF")

system.time(

for (i in 1:nmale) ### Group Loop

{

######## normalising gamma function ##############

#### un-normalised gamma function

prevalencegamma = function(x) {dgamma(x,shape=k_male[i],scale=theta_male[i])}

ncgamma1 = integrate(prevalencegamma, lower = 0, upper = 150) # str(ncgamma1) tells you it's a list of 5 and the value can be accessed via ncgamma1$value

#### normalised gamma function. This takes into account the proportion of drinkers compared to the total number of individuals in our population ####

prevgamma=function(x) {(1-(prop_abs_male[i] + prop_form_male[i]))*(1/ncgamma1$value)*dgamma(x,shape=k_male[i],scale=theta_male[i])}

#### filling the data frame with the group specific information

n_diseasemale = length(relativeriskmale)

AAFinfolistmale$REGION[(((i-1)*n_diseasemale)+1):(i*n_diseasemale)] <- inputmage$REGION[i]

AAFinfolistmale$SEX[(((i-1)*n_diseasemale)+1):(i*n_diseasemale)] <- inputmage$SEX[i]

AAFinfolistmale$AGE_CATEGORY[(((i-1)*n_diseasemale)+1):(i*n_diseasemale)] <- inputmage$AGE_CATEGORY[i]

#### Calculation of the AAFs for all disease categories ####

for (p in 1:n_diseasemale) ### Disease Loop

{

integral = function (x) {prevgamma(x)*(((relativeriskmale[[p]][[2]](x,relativeriskmale[[p]][[3]][1],relativeriskmale[[p]][[3]][2],relativeriskmale[[p]][[3]][3],relativeriskmale[[p]][[3]][4]))-1)) +1 )}

integralgamma = integrate(integral, lower = 0, upper = 150)

AAFgammamale=(prop_abs_male[i] + prop_form_male[i]*exp(relativeriskmale[[p]][[5]]) + integralgamma$value - 1)/(prop_abs_male[i] + prop_form_male[i]*exp(relativeriskmale[[p]][[5]]) + integralgamma$value)

##### creating a list of all information, the list contains: ####

##### filling the dataframe with the disease specific information

AAFinfolistmale$DISEASE[((i-1)*n_diseasemale+p)] <- relativeriskmale[[p]][[1]]

AAFinfolistmale$AAF[((i-1)*n_diseasemale+p)] <- AAFgammamale

}### loop p, representing the diseases

}###loop i, representing the different groups

) ### HF: end of system.time

##### Printing output and writing to file #####

##### Printing output and writing to file #####

print(AAFinfolistmale)

write.table(AAFinfolistmale, file="AAFmale-AGEGROUP1.txt")

################∞∞∞∞∞∞∞∞∞∞∞∞∞∞∞∞∞∞∞ COMPUTING THE CONFIDENCE INTERVALS ∞∞∞∞∞∞∞∞∞∞∞∞∞∞∞∞∞∞∞#################

################∞∞∞∞∞∞∞∞∞∞∞∞∞∞∞∞∞∞∞ COMPUTING THE CONFIDENCE INTERVALS ∞∞∞∞∞∞∞∞∞∞∞∞∞∞∞∞∞∞∞#################

################∞∞∞∞∞∞∞∞∞∞∞∞∞∞∞∞∞∞∞ COMPUTING THE CONFIDENCE INTERVALS ∞∞∞∞∞∞∞∞∞∞∞∞∞∞∞∞∞∞∞#################

################∞∞∞∞∞∞∞∞∞∞∞∞∞∞∞∞∞∞∞ COMPUTING THE CONFIDENCE INTERVALS ∞∞∞∞∞∞∞∞∞∞∞∞∞∞∞∞∞∞∞#################

###### Further improvement step: instead of computing a certain number of points we split

###### the code in m sets of nnn points

### m is the number of different sets of nnn points that will be computed

m=150

filenames=read.delim("filenames.txt", header=F, colClasses=c("character"))

for (t in 1:m)

{

################ The idea behind this section is the following: having realised how ################

################ difficult the expressions for the variances of our functions quickly ################

################ become, we will use the AAF function coded previously and apply it to ################

################ a set of randomly generated parameters. As we know the distributions of ################

################ our *first level* parameters, we can generate 10'000 points and apply ################

################ the whole algorithm to this set of parameters the evaluate the variance ################

################ of the AAFs at the end of it. ################

################ This has to be done in 2 steps. We first need to generate all the ################

################ parameters needed for the second one as some of them aren't straight ################

################ forward and have to be simulated too. ################

#### defining number of simulations, usually 10'000

nnn=1000

####∞∞∞∞ GENERATING PARAMETERS FOR MALE POPULATION ∞∞∞∞####

####∞∞∞∞ GENERATING PARAMETERS FOR MALE POPULATION ∞∞∞∞####

####∞∞∞∞ GENERATING PARAMETERS FOR MALE POPULATION ∞∞∞∞####

#### generating proportions of abstainers, former drinkers and drinkers. This is a binomial distribution considering a survey with 1'000 data points per sex-age group.

#### the output matrix is ordered in the following way: each line represents the 10'000 generated parameters for one group.

#### in order to compute the mean values we need the prop_abstainers for each region. To avoid 2 calculations these are combined together

prop_abs_listmale = NULL

prop_form_listmale = NULL

prop_drk_listmale = NULL

mean_male_list=NULL

mean_male_list_region=NULL

system.time(

for (i in 1:length(regions_male))

{

#### extracting the required information for each region ####

info_male_regionage = inputmage[inputmage$REGION==regions_male[i],]

info_male_region = inputm[inputm$REGION==regions_male[i],]

#### calculating the mean values for each group

population = info_male_region$POPULATION

coeffs=info_male_region$RELATIVE_COEFFICIENT

pcalitresperyear=info_male_region$PCA[1]

var_pca=info_male_region$VAR_PCA[1]

#### generating random values for pca

pca_listlitresperyear=rnorm(nnn, pcalitresperyear, sqrt(var_pca))

#### the following is to avoid having negative numbers as PCAs due to the random distribution.

for (h in 1:length(pca_listlitresperyear))

{

if (pca_listlitresperyear[h]<=0)

{

pca_listlitresperyear[h]=0.001

}

}

pca_list=0.8*pca_listlitresperyear*1000*0.789/365

mean_male_list_region=prop_abs_listmale_region=prop_form_listmale_region=NULL ### obviously, this has to be reinitialised at each region iteration

for (k in 1:length(info_male_region$AGE_CATEGORY)) #### this should usually be from 1 to 3

{

pabs=rnorm(nnn,info_male_region$LIFETIME_ABSTAINERS[k],sqrt(info_male_region$LIFETIME_ABSTAINERS[k]*(1-info_male_region$LIFETIME_ABSTAINERS[k])/1000))

pform=rnorm(nnn,info_male_region$FORMER_DRINKERS[k], sqrt(info_male_region$FORMER_DRINKERS[k]*(1-info_male_region$FORMER_DRINKERS[k])/1000))

#### It may happen that the generated proportions of abstainers and former drinkers will be larger than 1. In this case, we will set the propoprtion ####

#### of drinkers to 0 and scale the other 2 down. ####

for (h in 1:nnn)

{

if (pabs[h]+pform[h]>1)

{

sum=pabs[h]+pform[h]

pabs[h]=pabs[h]/sum

pform[h]=pform[h]/sum

}

}

prop_abs_listmale_region=rbind(prop_abs_listmale_region, pabs) ### this is a 3xnnn matrix used for the computation of the mu values below. These values are also stored in a greater matrix for further use in prop_abs_listmale

prop_form_listmale_region=rbind(prop_form_listmale_region, pform)

}

prop_abs_listmale=rbind(prop_abs_listmale, prop_abs_listmale_region[1,])

prop_form_listmale=rbind(prop_form_listmale, prop_form_listmale_region[1,])

for (j in 1:nnn)

{

prop_abs=prop_abs_listmale_region[,j]

prop_form = prop_form_listmale_region[,j]

mean_region = compute_mu(population[1],population[2],population[3],coeffs[1],coeffs[2],coeffs[3],prop_abs[1],prop_abs[2],prop_abs[3],prop_form[1],prop_form[2],prop_form[3],pca_list[j])

mean_male_list_region=cbind(mean_male_list_region,mean_region) ### this generates a 3xnnn matrix containing the mu values for each region, they will be placed in a bigger matrix mean_male_list

}

mean_male_list = rbind(mean_male_list, mean_male_list_region[1,])

}

) ### HF: system.time end

##### Generating the values for k and theta #####

ngroups = length (inputmage$AGE_CATEGORY)

k_list_male=NULL

for (i in 1:ngroups)

{

k_list_male_group = rnorm (nnn, (1/1.171)^2, sqrt(4*0.013^2/1.171^6)) # HF: 1 x nnn

k_list_male=rbind(k_list_male,k_list_male_group) # HF: ngroups x nnn

}

theta_list_male=NULL

for (i in 1:ngroups)

{

theta_list_male_group = mean_male_list[i,]/k_list_male[i,]

theta_list_male = rbind(theta_list_male, theta_list_male_group)

}

####### Generating the Betas for the Relative Risk Functions ##########

library("MASS")

#### male population ####

ndiseasesmale = length (relativeriskmale)

betacoefficients_male=list(rep(0,ndiseasesmale))

for (i in 1:ndiseasesmale)

{

betas = relativeriskmale[[i]][[3]]

covariance = relativeriskmale[[i]][[4]]

generatedbetas_disease = mvrnorm(nnn, betas, covariance)

betacoefficients_male[[i]]=t(generatedbetas_disease) #### we transpose only in order to have the values for each beta in a line and not a column

}

RRform_male_list=NULL

for (i in 1:ndiseasesmale)

{

lnRRform=rnorm(nnn,relativeriskmale[[i]][[5]],sqrt(relativeriskmale[[i]][[6]]))

RRform_male_list_disease = exp(lnRRform)

RRform_male_list=rbind(RRform_male_list,RRform_male_list_disease) ### this creates a vector ndiseasesmale X nnn -> each line corresponds to a disease and contains nnn occurences

}

##################∞∞∞∞∞∞∞∞∞∞∞∞∞∞∞∞∞ DEFINING THE AAF FUNCTION THAT WILL ITERATE THROUGH ALL THE POINTS ∞∞∞∞∞∞∞∞∞∞∞∞∞∞∞∞∞∞∞∞∞∞∞########################

##################∞∞∞∞∞∞∞∞∞∞∞∞∞∞∞∞∞ DEFINING THE AAF FUNCTION THAT WILL ITERATE THROUGH ALL THE POINTS ∞∞∞∞∞∞∞∞∞∞∞∞∞∞∞∞∞∞∞∞∞∞∞########################

##################∞∞∞∞∞∞∞∞∞∞∞∞∞∞∞∞∞ DEFINING THE AAF FUNCTION THAT WILL ITERATE THROUGH ALL THE POINTS ∞∞∞∞∞∞∞∞∞∞∞∞∞∞∞∞∞∞∞∞∞∞∞########################

#### for reasons of simplicity the code above to calculate the expected values of the AAF will not be implemented as a function. ####

#### however, this could be a further improvement of the code if there's more time ####

#### male population ####

#variances is the final table with all the AAFs and their confidence intervals

variances = data.frame(matrix(NA, nrow=length(regions_male)*length(agecategories_male)*length(relativeriskmale), ncol=6))

names(variances) = c("REGION","SEX","AGE_CATEGORY","DISEASE","AAF","VARIANCE")

system.time(

for (i in 1:nmale) ### Group Loop (sex/age/region)

{

##### improved code: for each iteration (of each region) we use a new table which is deleted at the end of the operation thus limiting the space used for the computation

AAFinfolistmalelist = data.frame(matrix(NA, nrow=nnn*length(relativeriskmale), ncol=5))

names(AAFinfolistmalelist) = c("REGION","SEX","AGE_CATEGORY","DISEASE","AAF")

niterations_male=length(relativeriskmale)*nnn

#### filling the data frame with the group specific information

n_diseasemale = length(relativeriskmale)

first_entry = 1

last_entry = n_diseasemale*nnn

AAFinfolistmalelist$REGION[first_entry:last_entry] <- inputmage$REGION[i]

AAFinfolistmalelist$SEX[first_entry:last_entry] <- inputmage$SEX[i]

AAFinfolistmalelist$AGE_CATEGORY[first_entry:last_entry] <- inputmage$AGE_CATEGORY[i]

for (z in 1:nnn) ## iterations loop (calculates nnn AAFs for each disease)

{

######## normalising gamma function ##############

#### un-normalised gamma function

prevalencegamma = function(x) {dgamma(x,shape=k_list_male[i,z],scale=theta_list_male[i,z])}

ncgamma1 = integrate(prevalencegamma, lower = 0, upper = 150,stop.on.error=FALSE)

if(ncgamma1$message=="OK")

{

#### normalised gamma function. This takes into account the proportion of drinkers compared to the total number of individuals in our population ####

prevgamma=function(x) {(1-(prop_abs_listmale[i,z] + prop_form_listmale[i,z]))*(1/ncgamma1$value)*dgamma(x,shape=k_list_male[i,z],scale=theta_list_male[i,z])}

#### Calculation of the AAFs for all disease categories ####

for (p in 1:length(relativeriskmale)) ### Disease Loop

{

integral = function (x) {prevgamma(x)*(relativeriskmale[[p]][[2]](x,betacoefficients_male[[p]][1,z],betacoefficients_male[[p]][2,z],betacoefficients_male[[p]][3,z],betacoefficients_male[[p]][4,z]))}

integralgamma = integrate(integral, lower = 0, upper = 150,stop.on.error=FALSE)

if (integralgamma$message=="OK")

{

AAFgammamale=(as.numeric(prop_abs_listmale[i,z]) + as.numeric(prop_form_listmale[i,z])*as.numeric(RRform_male_list[p,z]) + integralgamma$value - 1)/(as.numeric(prop_abs_listmale[i,z]) + as.numeric(prop_form_listmale[i,z])*as.numeric(RRform_male_list[p,z]) + integralgamma$value)

}

else

{

AAFgammamale=0

}

##### filling the dataframe with the disease specific information

AAFinfolistmalelist$DISEASE[((z-1)*n_diseasemale+p)] <- relativeriskmale[[p]][[1]]

AAFinfolistmalelist$AAF[((z-1)*n_diseasemale+p)] <- AAFgammamale

niterations_male=niterations_male-1

print(c("#of iterations left for male population: ",niterations_male,"for region", i, "from set #",t))

}### loop p, representing the diseases

}

else

{

### in case the first integral didn't work (prevalence assumed to be zero everywhere) we need to fill the corresponding AAF line with zeros

for (p in 1:length(relativeriskmale)) ### Disease Loop

{

AAFgammamale=0

AAFinfolistmalelist$DISEASE[((z-1)*n_diseasemale+p)] <- relativeriskmale[[p]][[1]]

AAFinfolistmalelist$AAF[((z-1)*n_diseasemale+p)] <- AAFgammamale

niterations_male=niterations_male-1

print(c("#of iterations left for male population: ",niterations_male,"for region",i,"from set #",t))

}### loop p in the case where the first integral didn't work

}## end of is/else statement for the first integral

} ###loop z, representing the different simulations of each group

#### Now we evaluate the CI for the age/sex/region under test and store the value in the final table

write.table(AAFinfolistmalelist, file=toString(c("AAFoutputs for ", regions_male[i], "of set ", t)) ,sep=",")

AAFlist_region=AAFinfolistmalelist[AAFinfolistmalelist$REGION==toString(regions_male[i]),]

for (j in 1:length(agecategories_male))

{

AAFlist_regionage=AAFlist_region[AAFlist_region$AGE_CATEGORY==agecategories_male[j],]

for (k in 1:length(relativeriskmale))

{

AAFlist_regionagedisease=AAFlist_regionage[AAFlist_regionage$DISEASE==toString(relativeriskmale[[k]][1]),]

varregagedisease=var(as.numeric(AAFlist_regionagedisease$AAF))

### finding the AAF corresponding to this CI in the previously obtained matrix

AAFreg=AAFinfolistmale[AAFinfolistmale[,1]==toString(regions_male[i]),]

AAFregage=AAFreg[AAFreg[,3]==as.numeric(agecategories_male[j]),]

AAFregagedis=AAFregage[AAFregage[,4]==toString(relativeriskmale[[k]][1]),]

AAF=AAFregagedis$AAF

#### creating the entry for the final list

entry <- ((i-1)*length(agecategories_male)*length(relativeriskmale) + (j-1)*length(relativeriskmale) + k)

variances$REGION[entry] <- regions_male[i]

variances$SEX[entry] <- AAFlist_regionagedisease$SEX[1]

variances$AGE_CATEGORY[entry] <- agecategories_male[j]

variances$DISEASE[entry] <- toString(relativeriskmale[[k]][1])

variances$AAF[entry] <- AAF

variances[entry,6] <- varregagedisease

}

}

}###loop i, representing the different groups

) ### HF: system.time end

print(variances)

write.table(variances, file=filenames[t,1],sep=",")

} ### t Loop for the different files
